# TALEN-mediated *Nanog* disruption results in less invasiveness, more chemosensitivity and reversal of EMT in Hela cells

**DOI:** 10.18632/oncotarget.2298

**Published:** 2014-07-31

**Authors:** Yan Ding, Ai Qing Yu, Cheng Lin Li, Juan Fang, Yi Zeng, Dong Sheng Li

**Affiliations:** ^1^ Hubei Key Laboratory of Embryonic Stem Cell Research, Taihe Hospital, Hubei University of Medicine, Shiyan, Hubei, China; ^2^ College of Life Science and Bioengineering, Beijing University of Technology, Beijing, China

**Keywords:** Nanog, Hela cell, cervical cancer cell, TALEN, epithelial-mesenchymal transition

## Abstract

Emerging evidence suggests that *Nanog* is involved in cervical tumorigenesis. However, the regulating role of *Nanog* in tumorigenesis and chemosensitivity are still poorly understood. In this study, *Nanog* was disrupted by transcription activatorlike effector nucleases (TALEN) in Hela cells and its expression was significantly decreased in a single-cell derived sub-clone with biallelic mutations. The disruption of *Nanog* not only induced down regulation of some other core transcription factor genes for cell self-renewal, such as *Oct4*, *Sox2* and *FoxD3*, but also led to the down regulation of some mesenchymal representative genes, *vimentin* and *N-adherin*, and up regulation of the epithelial gene, *E-cadherin*. In addition, the invasiveness and clonogenicity of the Hela cells were obviously affected, and surprisingly their sensitivities to anti-cancer drugs were also significantly increased *in vitro*. After Xenograft into nude mice, the growth volumes of the neoplasms from the *Nanog* disrupted Hela cells were significantly smaller compared with those from wild type ones. In conclusion, these results suggest that disruption of *Nanog* may reverse the status of epithelial-mesenchymal transition, which is critical in tumorigenesis, and alleviate chemoresistance, as well as their invasiveness, in cervical cancer cells.

## INTRODUCTION

The cervical cancers are the third most common cancers in females, with their mortality in the fourth place [1]. Human papilloma virus (HPV) is believed to play the major role in the etiology of these cancers, though some other factors may also be involved [2]. Poor prognosis is usually associated with the characteristics of highly invasive and diffusely metastatic [3]. In the initial stage of invasion and metastasis, the morphogenetic changes due to conversion of polarized epithelial cells to motile mesenchymal cells, are referred as epithelial-mesenchymal transition (EMT). However, how cervical cancer cells acquire the ability to invade surrounding tissues and metastasize is far from understood and little is known about the reversal of EMT in these tumors.

*Nanog* is a core transcription factor gene for maintaining self-renewal and pluripotency of embryonic stem cells [4, 5]. It has been reported that abnormal expression of *Nanog* is associated with human germ cells and several other types of cancers, such as gliomas [6], embryonic carcinomas [7], prostate cancers [8], breast cancers [9], etc. Down regulation of *Nanog* inhibits the proliferation, colony expansion and clonogenic growth of tumor cells [10], while over expression of *Nanog* may induce chemoresistance in breast cancer and prostatic cancers [11]. However, the correlation between *Nanog* expression and the cervical cancer remains unclear and the molecular mechanisms of *Nanog* in inducing EMT, metastasis and chemoresistance also need to be further clarified.

Since their discoveries, various kinds of RNAi techniques have been widely used to repress some gene expression *in vitro* because of their convenience in use. However, none of these techniques are able to induce complete disfunction of the target gene. The recently developed genome-editing techniques, such as transcription activator-like effector nucleases (TALEN), could overcome this problem [12]. TALEN could induce some mutations at random to a specific gene, which often lead to translational termination and loss of its function [13]. In the present study, *Nanog* of Hela cells was biallelicly disrupted by TALEN in order to investigate if it plays any role in affecting invasiveness, EMT and chemoresistance in human cervical cancers.

## RELSULTS

### T7E1 analysis and selection for cell clones with biallelic mutations

**Figure 1:**
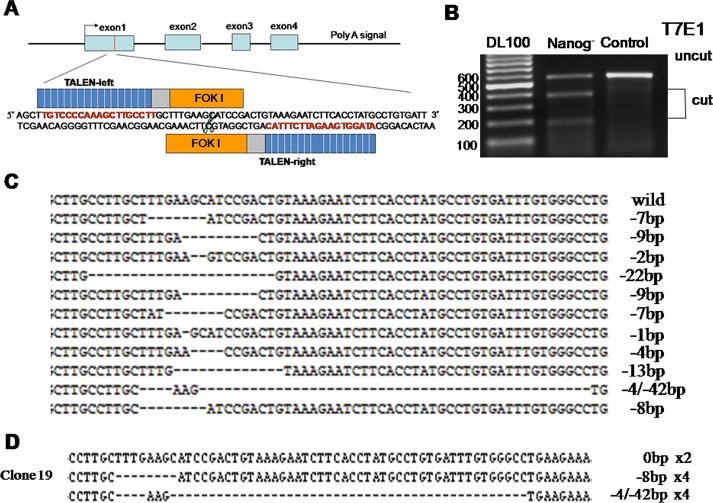
Disruption of *Nanog* in Hela cells by TALEN A: Diagrammatic sketch of *Nanog* and its target site of by TALEN plasmids. B: After two rounds of targeting by TALENs, the target sites of *Nanog* were amplified by PCR and then digested by T7E1 to evaluate the proportion of the mutated *Nanogs*. C: Alignment of the sequences of mutated and wild type *Nanogs* at the TALEN target sites. The number of deleted nucleotides (dashes) was marked on the right end of each sequence. D: Sequencing of *Nanog* for the selected cell clone after two rounds of targeting by TALEN. This result suggests there be at least three *Nanog* alleles in Hela cells. Control stands for the Hela cells of wild type; Nanog- stands for those with *Nanog* disruption.

To assess the endonuclease-dependent genome editing activities, gDNA were prepared from the transfected Hela cells after two rounds of transfection by TALEN. The target region of *Nanog* gene was amplified by PCR, and then digested by T7E1 enzyme. In result, TALEN induced *Nanog* mutations were found to be more than 50% and one cell clone was selected as with biallelic *Nanog* mutations by gene sequencing from 30 single-cell cultures (Figure 1). As a matter of fact, this biallelic *Nanog* disrupted Hela cell clone was not true *Nanog* knockouts due to the polyploidy nature of Hela cells [14, 15]. Obviously, there are at least three *Nanog* alleles in the selected Hela cell clone based on the gene sequencing results. Nevertheless, they were used for the following experiments.

### *Nanog* disruption alleviated the invasiveness of the Hela cells

**Figure 2:**
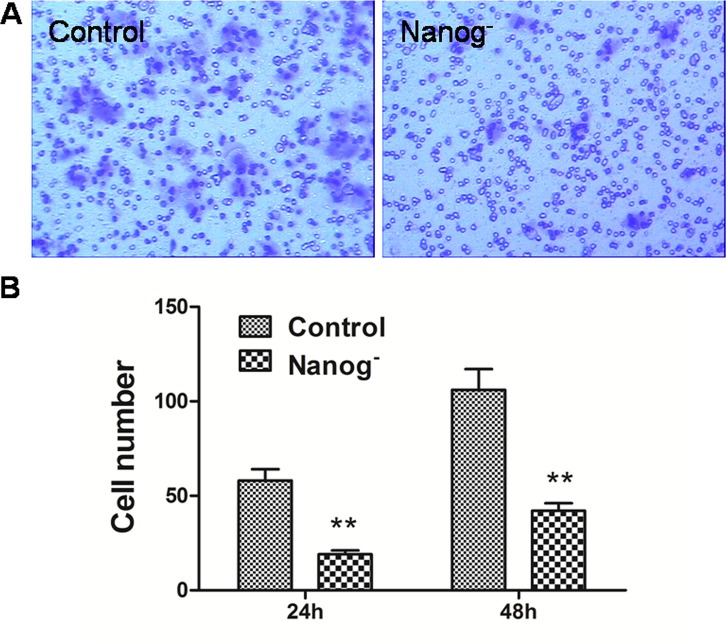
Transwell assay of the Hela cells with/without *Nanog* disruption A: the pictures taken by the imaging system. B: Comparison of the cell numbers that passed the membrane of transwell at 24h and 48h between the Hela cells with/without *Nanog* disruption. The differences were significant at the both time points. Control stands for the Hela cells of wild type; Nanog- stands for those with *Nanog* disruption.

**Figure 3:**
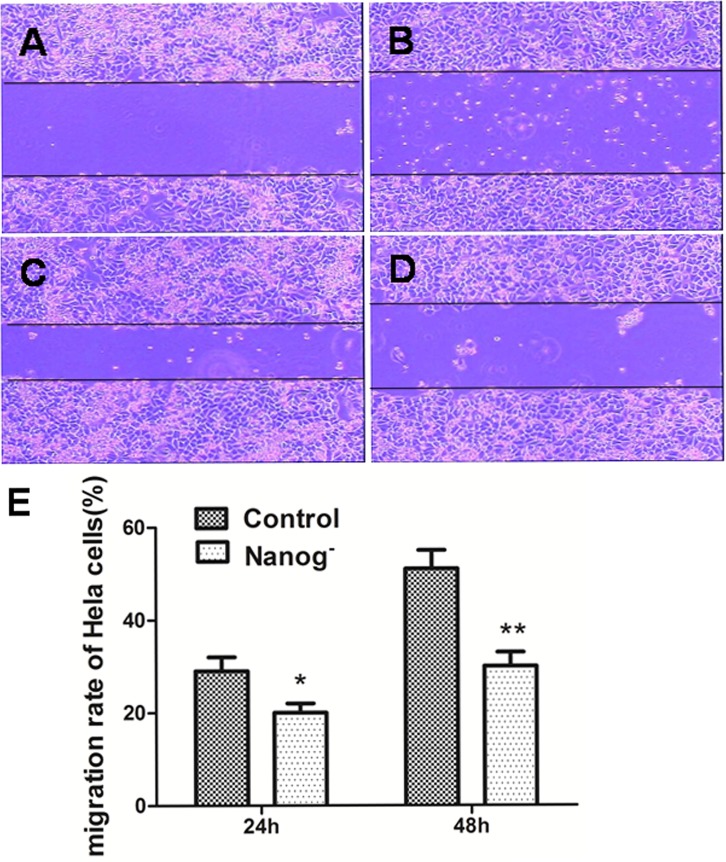
Scratch assay of the Hela cells with/without *Nanog* disruption A and C: The pictures taken at 0h and 24h for the wild type Hela cells. B and D: The pictures taken at 0h and 24h for the Hela cells with *Nanog* disruption. E: Comparison of migration distances between the Hela cells with/without *Nanog* disruption at 24h and 48h. The differences were significant both at 24h (*P<0.05) and at 48h (**P<0.01). Control stands for the Hela cells of wild type; Nanog- stands for those with *Nanog* disruption.

Transwell cell migration assay and scratch assay were carried out to investigate how the invasiveness of Hela cells was affected by *Nanog* disruption. As shown in Figure 2, the numbers of the cells that passed through the matrigel and transwell at 24h and 48h were 19±3 and 42±5 respectively for *Nanog* disrupted Hela cells, and 58±7 and 106±13 respectively for those with wild type *Nanog*. The numbers for *Nanog* disrupted Hela cells were remarkably less (P<0.05). In scratch assay, the migration rates for the *Nanog* disrupted Hela cells at 24h and 48h were 20±4% and 31±4% respectively while those for wild type *Nanog* Hela cells were 29±4% and 51±7% respectively (P<0.05, Figure 3).

### *Nanog* disruption decreased the cloning efficiency of the Hela cells

**Figure 4:**
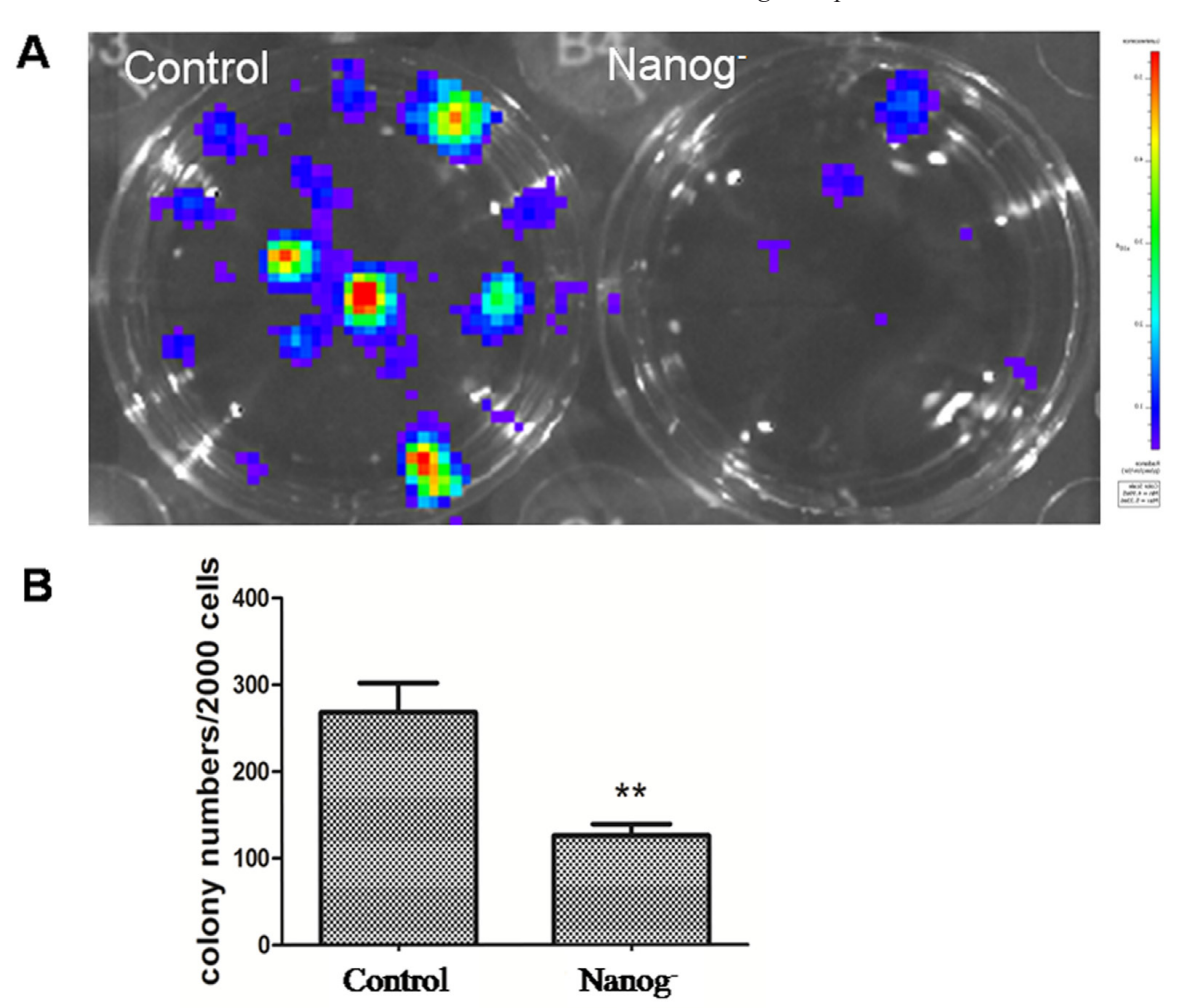
Clonogenicity assay A: The picture of the cell clones formed from the dissociated Hela cells with/without *Nanog* disruption after 10 days of incubation at the density of 1000 cells/well in a 12-well plate. B: Comparison of the cell colony numbers formed from dissociated Hela cells with/without *Nanog* disruption after 12 days of incubation. The colonies formed were significantly fewer for the Hela cells with *Nanog* disruption (**P<0.01). Control stands for the Hela cells of wild type; Nanog- stands for those with *Nanog* disruption.

In cell colony formation assay as shown in Figure 4, the colony numbers formed by *Nanog* disrupted Hela cells were 133±15, while those formed by wild type *Nanog* Hela cells were 285±32. This significant difference (P<0.05) indicated that the clonogenicity of Hela cells was greatly inhibited by *Nanog* disruption.

### *Nanog* disruption increased the chemosensitivity of the Hela cells

**Figure 5:**
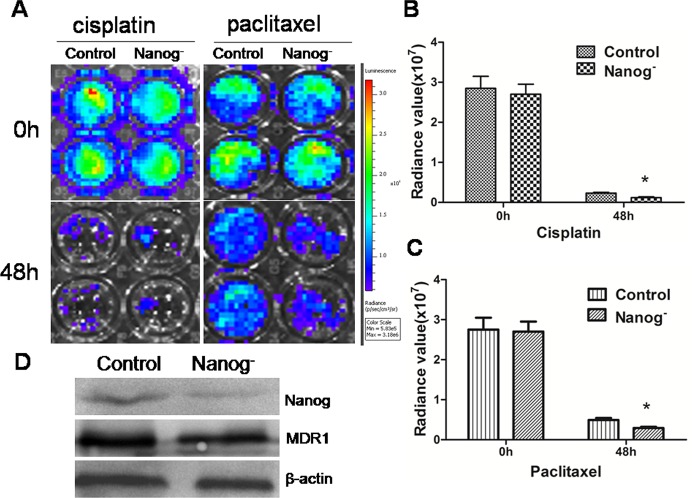
Chemosensitivity assay The Hela cells with/without *Nanog* disruption were exposed to cisplatin (1 μg/ml) and paclitaxel (40 ng/ml) separately for 48h. The cell viabilities were checked with a florescent imaging system. A: The picture by the imaging system showed that the cell viabilities were decreased more after exposed to both cisplantin and paclitaxel. B and C: Comparisons of the cell viabilities after exposed to cisplatin and paclitaxel respectively. The viabilities of the Hela cells with *Nanog* disruption were significantly decreased (*P<0.05). D: Western blot result showed that the level of MDR1 proteins of the *Nanog* disrupted Hela cells was decreased compared with that of wild type ones. Control stands for the Hela cells of wild type; Nanog- stands for those with *Nanog* disruption.

To evaluate the effect of *Nanog* disruption on chemosensitivity of Hela cells, both wild type and *Nanog* disrupted Hela cells were exposed to cisplatin or paclitaxel. In result, *Nanog* disrupted Hela cells were more sensitive to cisplatin and paclitaxel as shown in Figure 5A, B. The expression of MDR1, which is regarded as an important indicator for drug resistance in chemotherapy, was also significantly down regulated in the *Nanog* disrupted Hela cells (P<0.05).

### *Nanog* disruption reversed the status of EMT

**Figure 6:**
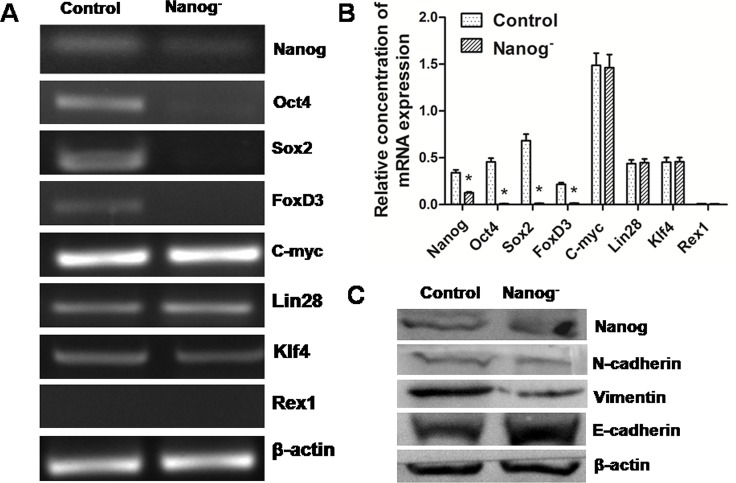
Reversal of EMT in the *Nanog* disrupted Hela cells A and B: Real-time PCR results showed that some self-renewal related transcription factor genes such as *Oct4*, *Sox2* and *FoxD3* were down regulated by *Nanog* disruption. C: Western blot results showed that after the disruption of *Nanog*, the expression of one epithelial marker, E-cadherin, increased, while that of two mesenchymal markers, N-cadherin and Vimentin, was decreased. These results strongly suggest that *Nanog* disruption could induce the reversal of EMT in Hela cells. Control stands for the Hela cells of wild type; Nanog- stands for those with *Nanog* disruption.

EMT is usually regarded as a progression sign toward malignancy. However, this progress seemed to be reversed by *Nanog* disruption. Apart from *Nanog*, several other genes, like *Oct4*, *Sox2* and *FoxD3*, were also remarkably down regulated in real-time PCR, though the expressions of *c-Myc*, *Lin28*, *Klf4* and *Rex1* remained unchanged (Figure 6A, B). Meanwhile, E-cadherin expression increased while that of vimentin and N-Cadherin decreased in Western blots (Figure 6C). All these changes suggested a reversal of EMT.

### The *Nanog* disrupted Hela cells grew slower in the Xenograft test

**Figure 7:**
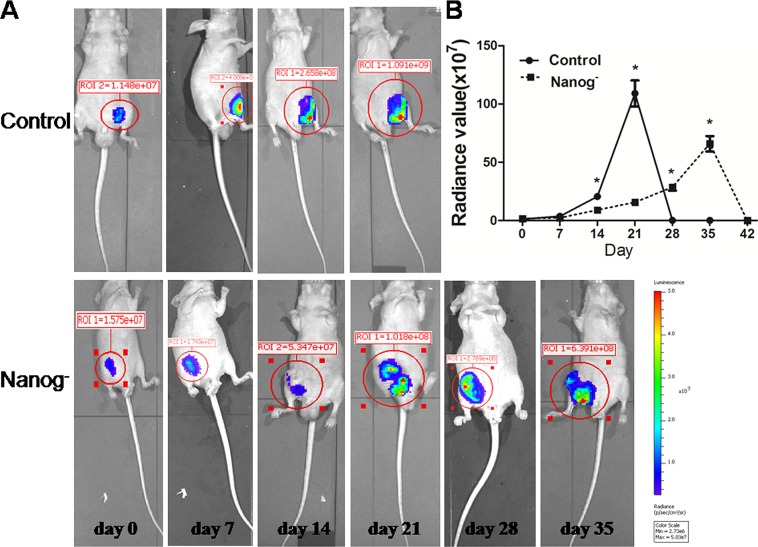
Xenograft test The Hela cells with/without *Nanog* disruption (10 million each) were subcutaneously injected into the flanks of nude mice (n=3) and the sizes of neoplasms formed were measured with an *in vivo* imaging system until mice died (usually 6~8 week). A: Serial pictures taken at different time points; B: The changes of fluorescein radiance values of the neoplasms formed from the injected Hela cells with/without *Nanog* disruption. The difference was significant (* P<0.05). Control stands for the Hela cells of wild type; Nanog- stands for those with *Nanog* disruption.

Both *Nanog* disrupted and wild type Hela cells were separately injected subcutaneously into nude mice and the sizes of neoplasms formed from implanted Hela cells were measured weekly. In result, the size of the neoplasms formed from *Nanog* disrupted Hela cells was significantly smaller than those from the wild type ones (P<0.05). Furthermore, the mice implanted with *Nanog* disrupted Hela cells lived longer than those with wild type ones (6-8 weeks vs. 4-5 weeks) (Figure 7).

## DISCUSSION

The development of a malignant tumor is a complex process of multiple factors and stages, involving malfunctions or mutations of a variety of genes. The EMT is believed to be one of the most critical steps for neoplasms to progress toward malignancy. It endows tumor cells with migratory and invasive properties [16]. Understanding the molecular mechanism underlines EMT and finding a way to stop or reverse it should be very important for developing new therapeutic strategies against various cancers.

In this study, *Nanog* was disrupted by TALEN and that seemed to result in a dramatic reversal of EMT process in Hela cells. Not only the expression of *Nanog* was remarkably inhibited, but also the expressions of some other core transcription factor genes for cell self-renewal, such as *Oct4, Sox2* and *FoxD3*, were down regulated. E-cadherin mediates epithelial cell-to-cell adhesion [17]. The decrease of E-cadherin expression is a critical step in the process of EMT [18], and it is often companied with increase of N-cadherin expression. Vimentin is one of the major mesenchymal intermediate filaments and its expression represents the completely dedifferentiated state in tumor cells that are highly proliferative and invasive [19]. Remarkably, the E-cadherin expression increased while the expressions of N-cadherin and vimentin decreased after disruption of *Nanog*. These data suggest *Nanog* may play a critical role in the process of EMT.

The grade of malignancy is closely related with the invasiveness and the clonogenicity of a tumor. Both transwell cell migration assay and scratch assay showed that the invasiveness of the *Nanog* disrupted Hela cells was significantly alleviated. Also, the clonogenicity of Hela cells was obviously affected in the cell colony formation assay. These findings suggested that the *Nanog* disrupted Hela cells become less malignant, which is very well in line with the phenomenon described above as a reversal of EMT process.

Chemotherapy has been widely used in the treatment of a variety of cancers including cervical cancers. However, the benefits are frequently limited by the chemoresistance of cancer cells through different signaling pathways, such as PI3K/AKT and MAPK (mitogen-activated protein kinase) [20, 21]. Studies have shown that the failure of chemotherapy in many malignant tumors was partially associated with abnormal expression of *MDR1* gene, which encodes the P-glycoprotein to pump anticancer agents out of the cells [22, 23]. Surprisingly in this study, *MDR1*, the most important indicator for chemoresistance in chemotherapy, was down regulated by *Nanog* disruption, though the mechanism underlined remained unclear. And these Hela cells did show more chemosensitivity when exposed to either cisplatin or paclitaxel.

In conclusion, our data suggested that the disruption of *Nanog* could reverse the status of EMT, which resulted in alleviation of invasiveness and poor clonogenicity in Hela cells, and that *MDR1*, an indicator gene for chemoresistance, could somehow be down regulated by even partial *Nanog* disruption, though it is usually thought to be regulated through other singling pathways, and this down regulation of *MDR1* did result in less chemoresistance.

## MATERIALS AND METHODS

### Cell Culture

The Hela cells transfected with a firefly luciferase gene (also named as Hela-luc cells) was a generous gift from Dr. Wang (University of British Columbia), were maintained in Dulbecco's modified Eagle's medium (DMEM) containing 10% fetal bovine serum (FBS).

### TALEN plasmids construction and cell transfection

The paired *Nanog* TALEN arms were designed according to the manufacture's instruction (Figure 1A). TALEN plasmids were constructed by one-step ligation using the Fast TALE^TM^ TALEN Assembly Kit (SIDANSAI).

About 10 μg of paired *Nanog* TALEN plasmids (each 5ug) were mixed with 1×10^6^ Hela cells in the cuvette with 100μl Opti-MEM. The transfection was carried out under 130 V by NEPA21 (Japan). The transfected cells were transferred into one well of 6-well plate and cultured at 37°C against puromycin at 3μg/ml. After 3 days, the cells were refreshed with the medium free of puromycin and kept at 37°C for few more days before harvested for DNA extraction and single-cell culture in order to select the *Nanog* disrupted cell clones.

### T7 endonuclease 1 (T7E1) assay

Genomic DNA was prepared from both the *Nanog* disrupted and wild type Hela cells with Blood Genomic DNA Extraction Mini Kit (TIANGEN). The genomic region encompassing the TALEN target site was amplified by PCR and its products were denatured and annealed to form heteroduplex DNA. The annealed DNA was treated with 5 units of T7E1 (Viewsolid Biotech) at 37°C for 15 min and then ran an agarose gel to separate the DNA fragments. The TALEN target rate was calculated on the intensities of DNA bands, which were proportionally measured by grey scale technique.

### Scratch assay

The scratch assay [2] was used to observe the cell migration. The Hela cells with/without *Nanog* disruption were cultured to completely confluent in 6-well plates and a scratch was made across the cell monolayer of each well with a 10μl pipette tip. Then washed the cell monolayer 3 times with D-PBS and incubated in serum free DMEM at 37 °C with 5% CO_2_ for 48h. The widths of the scratch were measured and the percentages of narrow down were compared at 0h, 24h and 48h respectively. The experiment was performed in triplicates.

### Cell migration assay

Cell migration assays were carried out with transwell filters as described by Liu et al [2]. Both the Hela cells with *Nanog* disruption and those without were digested and re-suspended with DMEM medium to 1×10^5^ cells/ml. 200μl cell suspension were seeded into an upper chamber of the polycarbonate membrane filter inserts with 8-μm pores (Corning, USA), which were pre-coated with matrigel. The lower chamber was filled with 500 μl of DMEM medium with 10% FBS. The cells (not migrated) in the upper chamber surface were removed with cotton swabs after incubated at 37°C for 24 and 48 h, and the cells (migrated) on the bottom side of the membrane were fixed with 95% ethanol for 30 min, stained with the 0.1% crystal violet and counted under a microscope. The experiment was performed in triplicates.

### Chemosensitivity assay

The chemosensitivities of the Hela cells with/without *Nanog* disruption were compared by the cell viabilities after exposed to cisplatin or paclitaxel. Briefly, after washed with PBS, freshly disassociated cells were seeded into a 96-well plate (1×10^4^ cells/well) and incubated at 37°C for 24h. Refreshed the cells with the growth media containing 1 μg/ml cisplatin or 40 ng/ml cisplatin and continually incubated for 48 more hours, took the cells out of the incubator, added 1ul luciferase substrate to the medium and left them at RM for 10-15 min, then checked cell viabilities with a florescent imaging system (IVIS ® Spectrum, caliper). The experiment was performed in duplicates and repeated three times.

### Clonogenicity assay

The clonogenicity was analyzed by colony-formation tests. The Hela cells with/without *Nanog* disruption were plated into 6 wells at 1000 cells/well in a 12-well plate pre-coated with 1% gelatin. The cells were incubated at 37°C for 10 to 12 days, until the cell colonies grew large enough to be visualized. Then the colonies were counted with the same imaging system as the chemosensivity assay used. The experiment was performed in triplicates.

### Xenograft test

The Hela cells with/without *Nanog* disruption were subcutaneously injected into nude mice (3 mice each group) separately. These mice were bred and the sizes of the neoplasms from implanted cells were checked weekly until the mice died with the same as the chemosensivity assay used.

### Quantitative PCR

The cells were harvested with TRIzol (Invitrogen) and total RNA was isolated according to the manufacturer's instructions. The cDNA were synthesized by using a reverse transcription kit (Takara Bio, China), and the quantitative PCR was conducted with a SYBR green one (Takara Bio, China). The PCR reaction proceeded as follows: 95°C for 30 sec, then 40 cycles including 95°C for 30 sec, 60°C for 30 sec and 72°C for 30 sec. The fold changes in gene expressions were normalized to β-actin. Gene-specific primers sets are shown in the Table 1.

**Table 1:** Primers used for RT-PCR

Gene	Sequence (forward; reverse)	Product length (bp)
*β-actin*	F: CTGGAACGGTGAAGGTGACA	140
	R: AAGGGACTTCCTGTAACAACGCA	
*Nanog*	F: CTCTCCTCTTCCTTCCTCCAT	104
	R: TTGCGACACTCTTCTCTGC	
*Oct4*	F: GACAACAATGAGAACCTTCAGGAGA	193
	R: TTCTGGCGCCGGTTACAGAACCA	
*Sox2*	F: CCCCCGGCGGCAATAGCA	215
	R: TCGGCGCCGGGGAGATACAT	
*Rex1*	F: CAGATCCTAAACAGCTCGCAGAAT	306
	R: GCGTACGCAAATTAAAGTCCAGA	
*c-Myc*	F: GATTCTCTGCTCTCCTCGAC	180
	R: TCCAGACTCTGACCTTTTGC	
*Klf4*	F: ACGATCGTGGCCCCGGAAAAGGA	392
	R: GTAGTGCTTTCTGGCTGGGCTCC	
*Lin28*	F: CCAGGCAAAAAGATCTGAAA	153
	R: AGAAAAGAGGGCAGGGTAGA	
*FoxD3*	F: GACGACGGGCTGGAAGAGAA	161
	R: GCCTCCTTGGGCAATGTCA	

### Western blot

Cells of each group were collected. The proteins were extracted and separated by SDS-PAGE, and then transferred onto PVDF membranes. After been blocked with 1% BSA for 1 h at room temperature (RT), the membranes were then incubated with antibodies against Nanog (1:200, Abcam), Vimentin(1:200, Santa Cruze) E-cadherin (1:200, Santa Cruze), N-cadherin (1:200, Santa Cruze), MDR1 (1:200, Santa Cruze), and β-actin (1:1000, TIANGEN) at 4°C overnight. After washing 3 times with TBST, the membranes were incubated with alkaline phosphatase-conjugated secondary antibody (1:500, TIANGEN) at RT for 1h. Then washed the membranes 3 times with TBST and imaged with a gel imaging system (BIO-RAD).

## STATISTICAL ANALYSIS

Results were presented as means of three independent experiments (M±SD). Statistical analyses were performed with the Student's t-test by using SPSS 13.0. P<0.05 was considered statistically significant.
